# Limiting data loss in infant EEG: putting hunches to the test

**DOI:** 10.1016/j.dcn.2020.100809

**Published:** 2020-06-26

**Authors:** Bauke van der Velde, Caroline Junge

**Affiliations:** a Experimental Psychology, Helmholtz Institute, Utrecht University, Utrecht, The Netherlands; b Developmental Psychology, Utrecht University, Utrecht, The Netherlands

**Keywords:** EEG, Data loss, Infants, Longitudinal, Data analysis

## Abstract

EEG is a widely used tool to study the infant brain and its relationship with behavior. As infants usually have small attention spans, move at free will, and do not respond to task instructions, attrition rates are usually high. Increasing our understanding of what influences data loss is therefore vital. The current paper examines external factors to data loss in a large-scale on-going longitudinal study (the YOUth project; 1279 five-month-olds, 1024 ten-months-olds, and 109 three-year-olds). Data loss is measured for both continuous EEG and ERP tasks as the percentage data loss after artifact removal. Our results point to a wide array of external factors that contribute to data loss, some related to the child (e.g., gender; age; head shape) and some related to experimental settings (e.g., choice of research assistant; time of day; season; and course of the experiment). Data loss was also more pronounced in the ERP experiment than in the EEG experiment. Finally, evidence was found for within-subject stability in data loss characteristics over multiple sessions. We end with recommendations to limit data loss in future studies.

## 1 Introduction

Infancy is a developmental period that marks clear changes in behavior and the brain. Electroencephalography (EEG) is an oft-used method to study these changes during infancy for two reasons. The high temporal resolution of EEG allows researchers to study closely linked brain-behavior correspondences. That is, to study whether and when infants can perceive contrasts between certain stimuli, researchers rely on changes in the EEG signal as a proxy for changes in behavior. Also, EEG can be used in an easy-going and non-threatening environment while infants are not required to make overt responses or to follow task instructions. Consequently, infant EEG research has a long history dating back to the 1930s when the development of sleep and awake EEG rhythms was studied in infants (Smith, 1938).

With the advance of neuroimaging techniques, the last decades saw an additional increase in studies relying on infant EEG (Azhari et al., 2020). However, measuring EEG in infants is not a straightforward task, and comes with its own challenges, often resulting in high attrition rates (Noreika et al., 2020; Stets et al., 2012). Fortunately, there is literature focusing on explaining and improving the methodology (cf. e.g., Bell and Cuevas, 2012; de Haan, 2007; Stets et al., 2012; Thierry, 2005). Our article aims to attribute to this literature by examining how factors beyond paradigm-specific parameters contribute to data attrition in infant EEG studies. Before we elaborate on the methodology of infant EEG, we will give a short overview of how EEG data can be used to better understand the developing brain.

Infant EEG researchers can derive several measures from the EEG signal. One of the most commonly used measures is the Event-related potential (ERP). In ERP paradigms, subjects are presented with certain types of stimuli numerous times while EEG is recorded. The ERP represents the averaged brain activity patterns to one type of stimuli within a short time window, beginning at the onset of a stimulus (‘time-locked’). Researchers then compare ERPs of different types of stimuli to understand whether infant brains can differentiate between different stimuli. With this paradigm, one can, for instance, observe whether and when infants can distinguish faces from other objects. This has been widely studied in adults, who show distinct differences in the peak around 170 ms after stimulus onset (N170) (Kanwisher et al., 1997). A similar peak is found in infants but slightly delayed after stimulus onset. Six-month-olds show differences in facial and object processing 290 ms after stimulus onset (Haan et al., 2002; Halit et al., 2004), which indicates that while infants do show distinct differences in the processing of faces versus other objects, it is not fully matured. This finding encapsulates the promise of using ERP as a behavioral proxy but is certainly not the only field for which ERPs have been used. ERPs have also been extensively used to study, among others, the development of language (Junge et al., 2012; Kuhl, 2010; Peña et al., 2010), emotions (de Haan et al., 2004; Leppänen et al., 2007), and joint attention (Kopp and Lindenberger, 2011; Striano et al., 2006).

The study of brain waves is a different EEG measure which specifically exploits the high temporal resolution of EEG (oscillations). Studying the oscillatory patterns of brain activity follows the hypothesis that synchronized oscillatory activity allows for an optimized flow of information between two regions (Fell and Axmacher, 2011). Therefore, it is likely that areas in the brain exhibiting similar oscillatory activity patterns are communicating or allowing for communication. These oscillatory activity patterns oscillate in functionally distinct frequency bands. For EEG, the frequency bands are (in order from low to high-frequency oscillations) the delta, theta, alpha, beta, and gamma frequency bands. In infants, EEG power analysis has been used to better understand, among others, the development of working memory (Bell and Wolfe, 2007), joint attention skills (Mundy et al., 2000), and motor development (Cuevas et al., 2014). More recently, EEG oscillatory information along with network analysis has been used to study the development of infant brain connectivity. Global network strength (the average connectivity in the whole brain), for example, has been related to autistic spectrum disorder symptoms: results show that infants who develop autism later in life exhibit higher global connectivity in theta and alpha frequency bands (Orekhova et al., 2014), while toddlers show lower global connectivity in the beta frequency band (Boersma et al., 2013). These global network measures are reliable in infants (van der Velde et al., 2019).

The aforementioned studies shortly sketch the breadth of possibilities in using EEG to study the infant brain and its development. One of the downsides in using EEG in infants, however, is the high rate of attrition. As it may be time-consuming and costly to recruit infants and their parents, high attrition rates might contribute to the fact that many infant (EEG) studies are underpowered (Bell and Cuevas, 2012; Frank et al., 2017; Noreika et al., 2020), increasing the likelihood of drawing false conclusions or resulting in non-replicable findings (Button et al., 2013). There are several reasons why attrition is high in studies testing infants. Awake infants cannot be instructed to remain attentive over the full course of an experiment, and the earlier the experiment is terminated before completion (because an infant will start crying, refuses to sit still, or falls asleep), the more likely an infant becomes excluded from further analysis. Of course, high attrition rates are found in any type of research involving awake infants (Frank et al., 2017; ManyBabies Consortium, 2020). However, EEG seems to have especially high rates of attrition even when compared to other infant study designs (Stets et al., 2012), with an average attrition rate of 49.2 percent based on 149 ERP articles published between 1990 and 2010.

This begs the question: what causes such high attrition factors in infant EEG studies? Ideally, future studies can use such information to minimize attrition rates during recording. Previous studies have aimed to shed some light on this question and determined several important factors that influence attrition rates. A recent meta-analysis comparing different paradigms on their attrition rates revealed that task-specific factors are a major influence, with auditory and audio-visual ERP studies resulting in markedly lower attrition rates than studies yielding visual ERP studies (Stets et al., 2012). Also, child characteristics partly explain the likelihood of attrition. For instance, infant temperament plays a role, with infants who are exhibiting more negative temperament showing higher rates of attrition (Marshall et al., 2009).

Additionally, Slaughter and Suddendorf found infant age to predict infant fussiness, with younger infants showing higher rates of fussiness, which they attributed to younger infants being more likely to fall asleep during the experiment (Slaughter and Suddendorf, 2007). While infant fussiness is a major reason to exclude infants in any kind of infant research, there is reason to believe that EEG recordings might place an additional burden on children’s willingness to complete the task: Some infants do not tolerate the designated headgear (net or cap) while other infants lose the required attention due to the repetitive nature of EEG paradigms (Slaughter and Suddendorf, 2007).

This additional burden on infants in the case of EEG causes trials to be contaminated by artifacts caused by movement, absence of data, or tiredness. To obtain a sufficient number of trials one solution might be to prolong experiments, thus anticipating data loss. However, ERP components can alter due to habituation processes (Stets and Reid, 2011). Moreover, it is important to note that there is no ‘golden standard’ in what the field considers the minimum number of clean trials required for a reliable. It varies from paradigm to paradigm and is also dependent on the variable of interest. For instance, there is evidence that an ERP-component related to visual attention (‘Nc-component’) is already visible in 7 trials (Stets and Reid, 2011), while a modulation of the ERP-component related to auditory processing (‘mismatch-negativity’) might require over 100 trials (Cheour et al., 1998). Therefore, prolonging experiments is not always ideal, and, as such, it is important to limit data loss from the onset in any experiment.

### 1.1 The current study: motivations and goals

This study aims to improve the process of infant EEG data collection by extending the literature on data attrition described in the previous paragraphs. Most of the previous work into origins of data loss compared attrition rates over various smaller studies, through (meta)-reviewing. In this study, we will focus on one large longitudinal study to examine factors that vary between individuals and which possibly contribute to data loss. This allows us to study relationships between factors that differ for each infant and their respective data loss. Some of these analyses aim to replicate previous findings while others serve to confirm researchers’ hunches to ideal circumstances of testing. To examine such effects, each factor needs to have enough variation to warrant further inspection. As such, large-scale studies are required since these studies invariably show high variability in testing conditions and environments, as keeping these steady over extended periods of time is impossible.

The large-scale study used here is the YOUth study (cf. Onland-Moret et al., this issue). YOUth fits our requirements for various reasons. It consists of two separate EEG-experiments – an ERP-study on face discrimination and a continuous video EEG-experiment on social versus non-social discrimination. Therefore, the YOUth study allows us to compare the effects of factors on attrition rates in two different tasks, one visual and one audio-visual, thus assessing whether any observed factor is viable across tasks (i.e., generalizable to other tasks) or whether it is task-specific. Additionally, the YOUth study is a longitudinal study, with infants visiting multiple times between the age of 5 months and 6 years. The study is on-going and aims to include 3000 children. At the time of writing, we have included 1279 five-month-olds, 1024 ten-months-olds, and 109 three-year-olds. This allows us to not only study the effects of a wide range of external factors on data loss but also enables us to assess whether longitudinal effects are working on attrition (e.g., some children are more prone to data loss than others).

This large-scale study will be used to determine whether data loss can be predicted based on several external factors. We use data loss as the dependent variable here, as the failure to meet the requirement to have a certain number of clean trials is one of the foremost reasons for attrition. What is important to note, however, is that high data quality does not equal low data loss. As mentioned earlier, in some ERP paradigms, a low amount of trials at the start of the experiment can provide similar or better results than when too many trials are used (Stets and Reid, 2011). Nevertheless, most experiments are constructed in such a way to yield a reasonable number of trials within a reasonable amount of testing. Of course, it remains questionable what is reasonable: there is no golden standard in the minimum number of trials. Nevertheless, at least for continuous EEG paradigms, the main assumption is that more clean data leads to higher reliability (Fraschini et al., 2016).

In the current paper, we examine various factors possibly related to data loss. Our factors of interest can be categorized into three groups. First, we focus on factors related to the infant, namely the gender, age, head shape, and the general well-being of the infant. The second group of factors related to the experimental conditions out of control of the subject: time of testing, the season of testing, whether the subject participated in other tasks before the EEG measurements, and research assistant (RA) present. Finally, as this study has a longitudinal design, we examined the stability of some data loss measurements within-subjects, namely the likelihood of data loss and attrition across visits.

In short, this paper is meant to illustrate the impact of a range of factors on data loss in infant EEG paradigms. Some of these factors of interest have been put forward as ‘hunches’ based on our own (subjective) experience but have never been put to a test: for example, the effect of season and time slot of testing. Other factors have already been proven in the literature, and we aim here to replicate them. Please note that our study is by no means meant as a complete overview of all factors possibly influencing infant EEG data loss. Moreover, although we will be analyzing two separate paradigms, one visual and one audio-visual, we do not know how well these results generalize to other studies, paradigms, locations, and setups. What we aim to achieve is to broaden our understanding of what factors could influence data loss. Therefore, this paper could prove useful for both novel researchers venturing into the world of infant EEG and experienced EEG researchers. Both novel infant and experienced EEG researchers can use these findings to set up new studies, taking heed to here described influential data loss factors and trying to keep these factors optimal over the course of their study. Additionally, experienced researchers can use this paper to better understand the data loss issues likely influencing their own datasets to detect possible biases during analysis.

## 2 Methods

### 2.1 Participants

The YOUth study is a longitudinal cohort study consisting of two large cohorts differing in age range. The YOUth Baby & Child cohort follows infants from 20 to 24 weeks gestational age until the age of seven years while the YOUth Child & Adolescent cohort follows children from the age of 8 until the age of 16 years. Both behavioral and cognitive development is tracked through numerous tasks and methods (e.g. eye-tracking, EEG, MRI, questionnaires). The YOUth study was approved by the Medical Research Ethics Committee of the University Medical Center Utrecht and all participants’ parents provided written informed consent. A brief overview of the YOUth study including the measurements conducted at each timepoint is available from https://www.uu.nl/en/research/youth-cohort-study (cf. Onland-Moret et al., this issue).

The current study only uses data from the YOUth Baby & Child cohort, since this is the only cohort in which EEG was measured, in young children from 5 months onwards. In total, 1278 5-month-old infants, 1046 10-month-old infants, and 104 3-year-old toddlers were included. The lower amount of 3-year-olds is due to the fact that measurements for the latter have started only recently, and data for all waves is still on-going. Data of 3-year-olds are only included in the data loss comparison between waves. All other analyses are done with only the 5 and 10-month-old infants. As attrition and data loss are fundamental elements of our study design, no infants were excluded from our analysis. Table 1 and Table 2 show the demographic and attrition information for our study, for the EEG and ERP paradigm, respectively. Attrition due to fussiness was counted when the infant was excluded from the analysis for having too little (or no) data due to the infant being too tired or inattentive, started crying or moving too much, or refused to wear the cap. Note that for these tables we categorized infants in the attrition group using a conservative threshold (data loss over 75 percent), but that in the remainder of the paper data loss is used as a continuous variable. Attrition due to experiment(er) error was counted when the RA logged this or when the resulting data file was corrupted. Attrition rates are 27 % or even lower, which is somewhat below the expected range (Stets et al., 2012).

**Table 1:** Demographic and attrition information – Continuous EEG.

	Gender	Total	Attrition		Exp. Error		Fussiness		Age (in days)	
		N	N	%	N	%	N	%	mean	sd
5m		1278	342	26.8	41	3.2	301	23.4	166.7	23.4
	Boy	628	160	25.5	18	2.9	142	22.6	166.7	23.0
	Girl	650	180	27.7	23	3.5	157	24.2	166.7	23.8
10m		1046	240	22.9	46	4.4	194	18.5	315.7	24.4
	Boy	514	111	21.6	20	3.9	91	17.7	316.3	24.4
	Girl	523	127	24.3	26	5	101	19.3	315.2	24.4
3y		104	15	14.4	6	5.8	9	8.7	957.8	161.2
	Boy	51	9	17.6	3	5.9	6	11.8	954.5	168.9
	Girl	50	6	12.0	3	6	3	6	961.1	154.6

**Table 2:** Demographic and attrition information – ERP.

Wave	Gender	Total	Attrition		Exp. Error		Fussiness		Age (in days)	
		N	N	%	N	%	N	%	mean	sd
5m		1278	328	25.7	34	2.7	294	23	166.7	23.4
	Boy	628	154	24.5	14	2.2	140	22.3	166.7	23.0
	Girl	650	174	26.8	20	3.1	154	23.7	166.7	23.8
10m		1035	261	25.2	41	4	220	21.3	315.7	24.4
	Boy	514	128	24.9	19	3.7	109	21.2	316.3	24.4
	Girl	521	133	25.5	22	4.2	111	21.3	315.1	24.4
3y		101	10	9.9	3	3	7	6.9	957.8	161.2
	Boy	51	6	11.8	2	3.9	4	7.8	954.5	168.9
	Girl	50	4	8	1	2	3	6	961.1	154.6

### 2.2 Apparatus and stimuli

EEG was recorded using a cap with 32 electrodes (ActiveTwo system, BioSemi) positioned according to the international 10/20 system, at a sampling rate of 2048 Hz. A Common Mode Sense (CMS) and Driven Right Leg (DRL) electrode were used to provide an active ground. During the EEG recording, all infants and toddlers were seated 65 cm from a computer screen.

The presentations of the two experiments were in a fixed order. The first experiment was the ERP experiment in which children saw pictures of faces with neutral expressions and houses. Pictures were presented for 1000 ms, and the ISI was 700–1000 ms. There were 96 trials: 48 in the neutral face condition (4 × 12 models) and 48 in the house condition (4 × 12 houses). Order of stimuli was pseudo-randomized: per block of 24 trials (4 blocks in total), all pictures appeared once in a randomized order. Between blocks and whenever the infant was not looking at the screen, the experimenter played additional sounds or video clips as attention getters. The task lasted approximately 3−4 min.

The other experiment was a continuous EEG experiment, which consisted of two, one 1-minute long, videos repeated three times. One video depicted singing women, while the other depicted moving toys without human interference. In between videos, short breaks were taken (5 in total) after which the new video was started. Similar videos were used earlier in a study by Jones and colleagues (Jones et al., 2015). During both experiments, research assistants were allowed to pause the task if the child got too fussy. This task lasted 6−7 min. Tasks could be stopped at any time if either parent or child prevented continuation.

### 2.3 Data loss calculation

The calculation of data loss was similar for both tasks. EEG data were analyzed exclusively using MATLAB, using the FieldTrip toolbox (Oostenveld et al., 2011). The original 2048 Hz data were downsampled to 512 Hz, using chip interpolation and band-pass filtered at 0.1–70 Hz with a two-way Butterworth filter. A notch-filter at 50 Hz was used to remove the background mains hum. The common average was used as a reference. For the ERP task, epochs were created based on stimulus presentation which led to 96 one-second epochs cut from 200 ms before stimulus presentation until 800 ms after stimulus presentation. For the continuous EEG task, the data was divided into 360 equal one-second epochs.

Data loss calculation was kept as analogous as possible to the regular cleaning of EEG data. Trials were rejected based on the following criteria: amplitude (>+-250uV); jumps; kurtosis (>7); and absence of data. Jumps were detected using the FieldTrip toolbox^1^ . Thresholds were chosen based on commonly used thresholds in earlier EEG studies. However, both maximum amplitude and kurtosis are subjective thresholds (which researchers can disagree on). To prevent eventual influence of subjectivity in choosing thresholds on data loss calculation, we also calculated data loss with a wide range of thresholds, ranging from stringent (amplitude > +-100uV & kurtosis > 3) to lenient (amplitude > +- 300 & kurtosis > 8). Outside these ranges, almost all data were respectively removed or included, which makes determining differences between factors impossible. We did not observe any noticeable differences depending on our choice of rejection criteria: results were similar regardless of whether we used more stringent or more lenient thresholds. This is not surprising as correlations between data loss values found for each subject for different leniency in data loss calculation methods were high (0.83 < r < 0.91). We, therefore, decided to maintain our relatively lenient thresholds for artifact rejection.

**Fig. 1:**
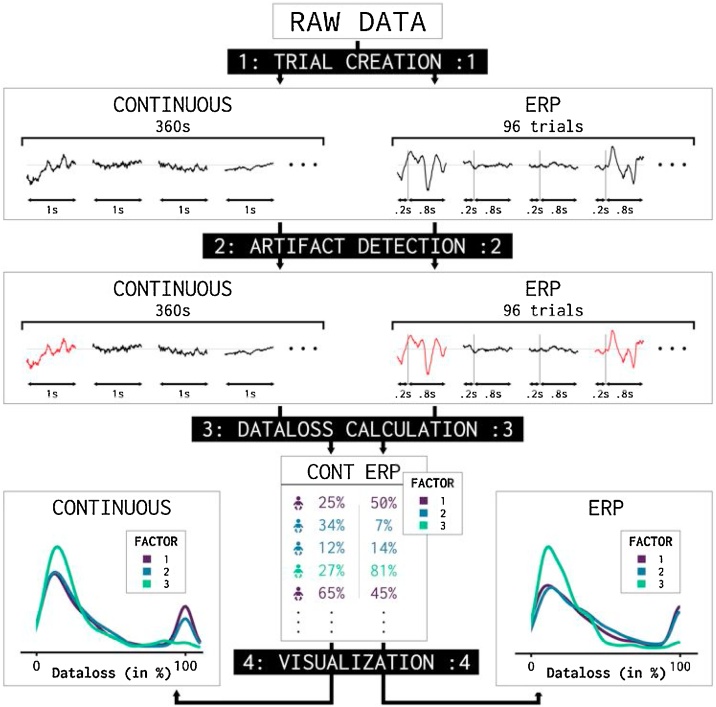
Graphical overview of analysis design. Data is analyzed in four steps. 1) Raw data is cut into 1 s trials for both the continuous and ERP task, resulting in 360 and 96 trials respectively. 2) Jump, noise and flatline artifacts are detected and trials containing artifacts are selected. 3) Data loss is calculated by calculating the percentage of trials containing artifacts over the total expected trials (360 in the case of the continuous experiment and 96 in the case of the ERP experiment). 4) Subjects are grouped based on factor and the data loss distributions are visualized using a probability density function. The probability density functions plotted in the visualization step are made using the gramm toolbox (Morel, 2018). The stat_density.m function is based on the standard ksdensity.m function in matlab.

All trials with artifacts based on the criteria mentioned above in any channel were counted as bad trials (N_badtrials_). Data loss (DL) was calculated as the percentage of bad trials of all expected trials separate for the ERP and the continuous experiment with the following formula:

$$DL=\;\frac{N_{badtrials}}{N_{expectedtrials}}*100$$

with N_expectedtrials_ = 96 in the case of the ERP task and N_expectedtrials_ = 360 in the case of the continuous task. Channels with more than 40 percent data loss were considered ‘bad channels’ and removed. Bad channels were interpolated using weighted averaged neighboring clean channels. Whenever there were more than two bad channels, we removed the entire subject was removed entirely, and set the data loss of that particular subject to 100 percent. Therefore, we expected two peaks in data loss distributions. One ‘low-data-loss’ peak for subjects who have successfully completed the experiment with limited to no data loss (around 20 percent data loss) and one ‘high-data-loss peak for subjects who showed little to no clean data (around 100 percent data loss). A graphical summary of the calculation of data loss is shown in Fig. 1.

### 2.4 Creating groups based on factors

Demographic information of all infants tested can be found in Table I. Since the number of infants tested per factor changes (number of infants tested by selected RAs are less than infants tested during a type of season), all number of infants per analysis are separately mentioned in the appropriate figures. For every analysis, infants were grouped according to factor. The following child-related factors were used to group infants:

Gender (for more info on demographics see Table 1 and Table 2).

1 Age. We grouped age by the wave as used in the YOUth project (5-month-old vs. 10-month-old vs. 3-year-old). Note that since we only recently started testing 3-year-old toddlers, the number of subjects is considerably lower (for more info on demographics see Table I and Table II).
2 Head shape. This was logged by the RA present during the testing day. There are four possible options: normocephaly (regular skull shape), brachycephaly (shorter than usual skull shape), plagiocephaly (skull with a flat spot), and scaphocephaly (elongated skull). Note that these are simplified denominations and RAs were only asked to group infants based on the category which best represented the infant’s head shape
3 General well-being of the infant. The RA asked the parent after testing whether the child was experiencing a typical day, or whether there was something amiss. There were three possible answers: “My child is having a typical day”, “my child is ill”, and “my child is tired / did not sleep well”

The following setting related factors were used to group infants:

1 Time of EEG experiment. We grouped these into four time slots: early morning testing (08:00−10:00), late morning testing (10:00 – 12:00), early afternoon testing (12:00−14:00), and late afternoon testing (14:00+). The latter was exceedingly rare and was therefore discarded from the study.
2 Order of testing during the test day. Besides the EEG-testing, the infant also took part in an eye-tracking session and a parent-child interaction session. Therefore, the EEG session could be the first, second, or third session (cf. Onland-Moret et al., this issue).

Season of testing: spring, summer, autumn, winter.

3 Research assistant (RA). Our approach is similar to the one employed by Hessels and Hooge (2019), who assessed the influence of RA in the YOUth cohort on data loss in the eye-tracking sessions for those RAs who tested at least 33 infants per wave. In our case, there were four RAs that tested clearly more infants than the others: these four (coded RA1, RA2, RA3, and RA4) tested over 40 5- and 10-month-old infants. In addition, to observe whether RAs improve with increasing experience, we tested the effect of time of RA on data loss over time for all RAs.
4 Task length. To study the influence of task duration on data loss, we logged the average amount of data loss as the task progressed. This allowed us to follow the progression of data loss and whether or not taking breaks in between trials played a positive role in limiting data loss throughout the experiment. Breaks in the continuous EEG experiment were breaks in between videos, during which a new video was started up. Breaks in the ERP experiment were videos used as attention grabbers shown every 24 trials.

For the following factors, stability over session was determined:

1 Attrition due to fussiness. As Table 1, Table 2 show for each wave, several children were to be excluded for further analysis, even though they participated in other sessions (i.e., eye tracking or parent-child interactions). In all cases, too little (or no) data was clean enough for analysis. Attrition due to fussiness of the infant was counted when the task was stopped either by the RA or parent, due to excessive movement, refusal to wear the cap, inattentiveness, sleepiness, or crying. When too much noise was detected in the data for analysis (either through too few trials surviving cleaning or more than 2 channels being noisy) and the infant was logged as restless or crying, this child was also to be counted as attrition due to fussiness.
2 Cap refusal. A subset of too fussy infants, but only those who specifically did not start the EEG-experiment but did participate in other experiments during the day.
3 Data loss. For those infants who participated in the tasks, we categorized them based on the proportion of data loss: a low group (the lowest 50 percent of data loss) and a high group (the highest 50 percent of data loss).

To prevent unreliable visual and statistical comparisons, only the categories of the categories within a factor which included more than 40 subjects were used for visualization and statistical analysis.

### 2.5 Data visualization and statistics

Data loss for infant and setting-related factors was visualized using a kernel-smoothed density plot, using MATLAB, with automatically determined bandwidth. The kernel-smoothing is used to increase the ease of visual comparisons between groups. Data is plotted using the gramm MATLAB toolbox (Morel, 2018). As mentioned earlier, data loss distributions have two distinct peaks: one around 20 percent data loss and another one around 100 percent data loss. Therefore, data is non-normally distributed, which is why we used non-parametric tests to compare groups. For this, we used the Kruskal-Wallis H test (Kruskal and Wallis, 1952), which is an extension of the Mann-Whitney *U* test (Mann and Whitney, 1947) and can be used for comparing two or more independent samples of equal or different sample sizes. To test the effect of experience on RA data loss, a linear regression was performed with experience as a running number of the number of infants tested by the RA. To test the stability of longitudinal factors over the course of the entire study, cross-tabulation was used to visualize results and chi-square tests of independence were used to determine correlations between the categorical variables.

## 3 Results

We carried out analyses and created figures for both the EEG and the ERP experiments. Most figures show distributions of data loss. The data loss of each infant is one value in each distribution. Higher amounts of data loss imply noisier data. Therefore, distributions with its center of gravity further towards the left signify generally cleaner data.

Since both tasks yielded similar results for most comparisons, we decided to present only the results for the continuous EEG task in the main article, as these results were determined using more trials. Only when there was a difference between the tasks we present both results in the main article. The results for the ERP task are in the supplementary materials.

### 3.1 Influence of child-related factors on data loss

**Fig. 2:**
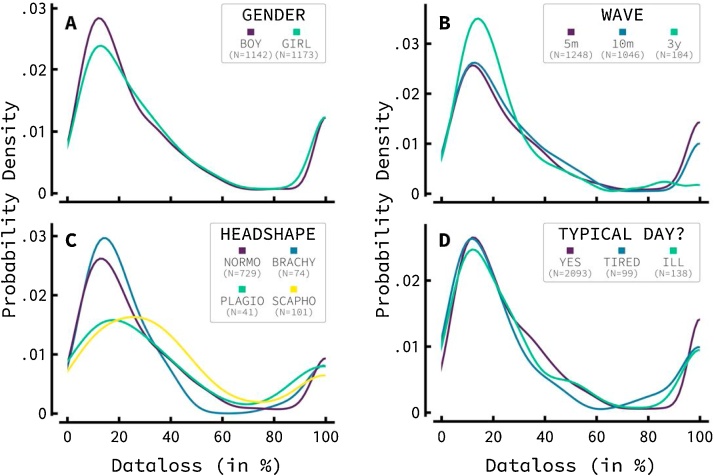
Influence of infant related factors on infant EEG data loss. This Figure shows probability density functions of data loss for different factors relating to infants. Note that all distributions show two distinct peaks. One at 100 percent, indicating all infants discarded from the data set, either due to very noisy data or due to too many bad channels; and one at +/- 15 percent indicating the group of infants who very successfully participated **A)** The data loss distributions for boys and girls: boys have a larger likelihood to have lower data loss. **B)** The data loss distributions between waves show a large effect for 3-year-old toddlers, who show markedly improved data loss. Data loss between 5-month-old and 10-month old waves is similar. **C)** Data loss distributions for different head shapes (normocephaly, brachycephaly, plagiocephaly, and scaphocephaly). Large differences can be seen in data loss distributions, with both plagiocephalic and scaphocephalic infants showing markedly higher and highly varying data amounts of data loss. **D)** Data loss distributions for ill or tired children showed no clear effect on data loss distributions compared to infants who participated during a typical day.

Distributions of data loss for four different factors regarding the child tested are shown in Fig. 2. Fig. 2A presents the different data loss distributions for different genders. Data loss was nearly identical across genders. For both genders, there are bimodal distributions of data loss, with clear peaks around 15 and 100 percent data loss. The peaks around 100 percent appear similar, while the early peak is more pronounced for boys. In other words, boys show a slight increase in lower amounts of data loss compared to girls. A Kruskal-Wallis H test using gender as a fixed factor resulted in a significant effect of gender at the p < .01 level (χ^2^(1) = 7.282, *p* = 0.007), indicating that boys have slightly lower data loss compared to girls.

Fig. 2B visualizes the data loss distributions for the different waves as used in the YOUth study. Infants in both the 5- and 10-month-old waves show very similar data loss distributions. The 3-year-old wave, however, revealed marked improvement. Three-year-old toddlers were less likely to be 100 percent discarded and more likely to show lower amounts of data loss. The fixed factor wave was tested using the Kruskal-Wallis H test, which found a significant effect at the p < .05 level (χ^2^(2) = 8.925, *p* = .012).

The data loss distribution for different head shapes of participating infants is depicted in Fig. 2C. Data loss distributions show distinct differences. The peak at 100 percent data loss is similar across head shapes. However, both normocephalic and brachycephalic infants show a higher likelihood of low data loss, with a higher peak of around 15 percent. Both plagiocephaly and scaphocephaly show peaks further to the right and highly variable amounts of data loss. This effect was tested to be significant at the p < .01 level (χ^2^(3) = 11.832, *p* = .008).

Lastly, Fig. 2D shows the effect of any subtle problems the infant might have had during testing according to the parent present. The parent was asked whether the child had been ill, was tired, or was experiencing a typical day. Both tiredness and illness showed no marked effect on data loss distributions. A Kruskal-Wallis H test yielded no significant results. ERP data showed similar results and are depicted in Supplementary Fig. 1.

### 3.2 Testing related factors on data loss

**Fig. 3:**
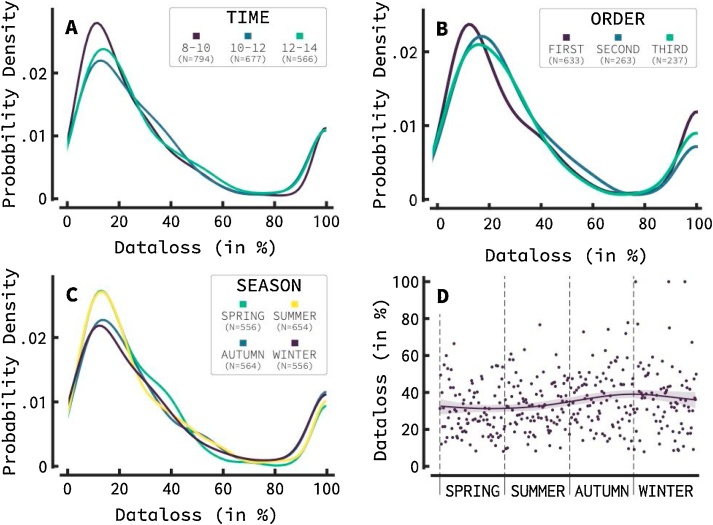
Influence of timing related factors on infant EEG data loss. This figure shows probability density functions of data loss for different factors relating to the timing of testing. **A)** shows that starting time influenced data loss, with early starting infants (between 8 a.m. and 10 a.m.) showing cleaner EEG data. The low data loss peak is slightly displaced to the left and a higher peak indicates a higher portion of infants has low data loss early in the morning. Time slots did not cause differences in infants with 100 percent data loss. **B)** shows that order of testing has limited influence on data loss: whether EEG was the first, second, or third task of the day, data loss was relatively similar. **C)** shows that season of testing also considerably influenced data loss. Infants showed lower data loss in sunny months in the Netherlands with a higher portion of infants showing low data loss and a lower portion of infants showing 100 percent data loss. **D)** shows the data loss of the study, averaged over year. Each dot represents the average data loss per day. A clear increase in data loss can be seen around late autumn / early winter. A smoothed line was drawn, which can be used as a visual aid (using Eilers and Marx’ method with automatic lambda (Eilers & Marx, 2002).

Fig. 3 shows distributions related to the timing of the experiment. As can be seen in Fig. 3A, data loss distributions were different across EEG timeslots. This is more visible in the peak resembling the lower data loss group: early EEG testing (between 8 a.m. and 10 a.m.) leads to a higher likelihood for the infant to be in the lower data loss group. The difference in timeslots is not apparent in the second peak, which resembles those infants with a 100 percent data loss. This suggests that the time of day only affects data loss for those infants who complete the tasks, but not for those infants for whom the task was terminated prior to completion. A Kruskal-Wallis H test with timeslot as fixed factor yielded a significant result at the p < .01 level (χ^2^(2) = 12.023, *p* = .002).

The influence of experiment order is presented in Fig. 3b. Recall that during the test day, each infant participates in three sessions in random order: the EEG-experiments, an eye-tracking session, and a parent-child interaction (PCI)-session. Each other session usually takes 10−20 min. To limit the influence of early morning testing, which is strongly correlated to EEG being the first experiment tested, only infants who had their EEG-experiment after 10 a.m. were taken into account. Fig. 3b shows that there is little difference in data loss distributions whether the infant participates in the EEG-experiment first, second, or last during a test day. The Kruskal-Wallis H test yielded no significant results. The ERP data showed similar results and are depicted in Supplementary Fig. 3.

Fig. 3C visualizes the effect of season of testing on data loss. Warmer seasons in the Netherlands (spring and summer) show markedly lower amounts of data loss compared to colder seasons (autumn and winter). This effect was visible in both peaks: in a higher amount of lower data loss infants and lower amounts of 100-percent data loss infants. The Kruskal-Wallis H test confirmed this effect to be significant at the p < .01 level (χ^2^(1) = 7.011, *p* = .008). Fig. 3D adds to this by showing the average data loss per day for every day in the year. A clear bump can be seen in the autumn and winter months. Supplementary Fig. 2 shows this effect throughout the entire study (so not averaged per year), with clear bumps in data loss at the start and end (winter and autumn) of 2016, 2017, 2018, and 2019, compared to spring and summer each year

**Fig. 4:**
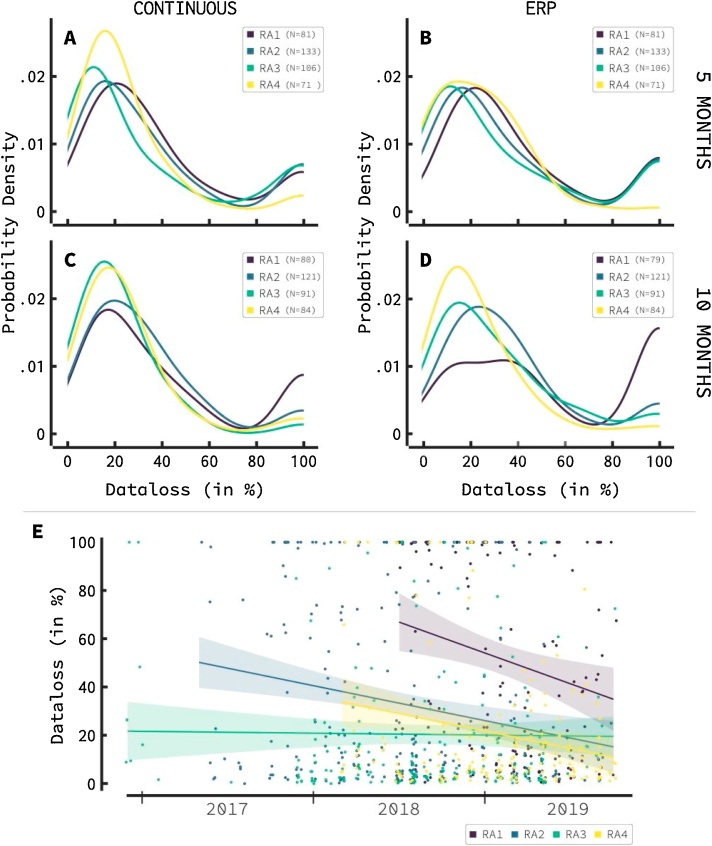
The influence of four research assistants (RAs) on data loss. Shown RAs have tested at least 40 infants in the 5 and 10-month waves in both continuous and ERP tasks. Clear influence of RA can be seen across all tasks and age groups. RA3 and 4 continuously outperform RA1 and 2, with low data loss peaks shifted to the left in both the continuous and ERP task. The proportion low data loss is also higher for RA1 and 2 in the continuous task and the ERP task with 10-month-old infants. The ERP task shows lower influence of RA in the 5-month-old wave compared to the 10-month-old wave. Ranking of assistants remains consistent over age groups and tasks (RA4 > RA3 > RA2 > RA1). E) shows the data loss per previously studied RA over time. A clear downward trend can be detected for three of the four studied RAs. Indicating an effect of experience on data quality per RA.

Fig. 4 shows data distributions across different research assistants (RAs). The only RAs included here are RAs who tested at least 40 infants of both 5-month-wave and 10-month-wave infants. The four panels show the distributions of data loss for infants in these waves (top and bottom), for both the continuous and the ERP task (left and right).

The continuous task (left panels) reveals large differences in data loss distributions between RAs, with RA 3 and RA4 outperforming RA1 and RA2 across age groups. Peaks of infants with low data loss can be seen further to the left. Clearly, RA4 has a distinctly lower 100 percent data loss peak compared to the other RAs. Kruskal-Wallis H tests were conducted, separately for each of the waves. The effect of RAs on the 5-month-old data loss was found to be significant at the p < .01 level (χ^2^(3) = 11.549, *p* = .009). A significant effect was also found for the effect of RAs on the 10-month-old data loss at the p<.001 level (χ^2^(3) = 20.207, *p* < .001).

The influence of RAs on data loss during the ERP task is shown in the right panels of Fig. 4. In contrast to the continuous EEG sessions, we note that there were only limited differences between RAs in the 5-month-old infants. However, clear differences can be seen at the 100 percent peak, with RA4 showing lower proportions of infants with 100 percent data loss. Also, a slight displacement can be seen in the low data loss infant peak with RAs 1 and 2 being slightly more shifted to the right. Differences in the 10-month-old wave are more distinct with once again RA3 and RA4 outperforming RA1 and RA2. Kruskal-Wallis H tests yielded significant results at the p < .01 level in the 5-month-old wave ERP data (χ^2^(3) = 13.268, *p* = .004) and at the p<.001 level in the 10-month old wave ERP data (χ^2^(3) = 40.984, *p* < .001).

What can be clearly seen from these distributions is that the ranking between RAs remains similar across tasks and waves (RA3 and 4 versus RA1 and 2), with RA4 outperforming all other RAs across tasks and waves, and RA1 performing worst across all tasks and waves. It is therefore likely that RA is the driving factor in data loss caused here, as RA performance appears consistent across waves and tasks.

Fig. 4E shows data loss for 5 and 10-month-old infants for each of the four RAs as a function of time across the years, capturing their experience. Clear effects of experience can be seen in three of the four RAs: data loss decreases over time. To test this the effect of experience a linear regression was calculated using RA experience of all RAs (not just the four RAs mentioned above) to predict data loss. The following significant regression equation was found (F(1,2199) = 10.849), p < 0.01, *R*^2^ = 0.005).

**Fig. 5:**
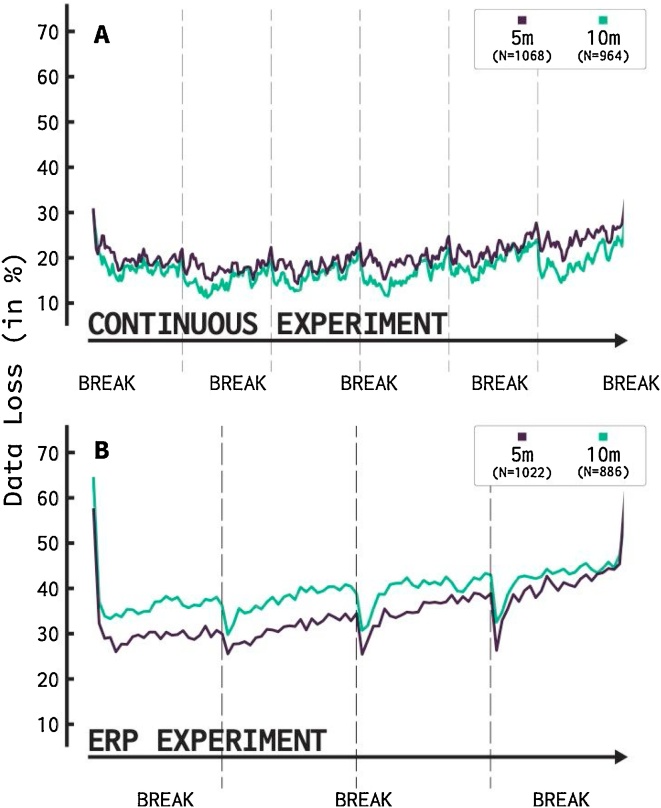
Data loss over the course of the entire experiment. A) The continuous experiment consists of 2 unique videos, repeated 3 times. After each video a short break is experienced during the starting up of the new video. Clear upward trends can be spotted over the course of each separate video. This upward trend is reset after each break. A slight upward trend over the course of the entire experiment can also be detected. Both 5 and 10-month-old infants showed similar data loss over time. B) The ERP experiment consists of 96 trials, with mandatory breaks after every 24 trials, during which a video is shown. Here also a clear effect of break can be seen, with stark decreases in data loss after each break, especially later in the experiment. Contrary to the continuous experiment, however, the improvement does not last for much longer than 2 trials, which indicates that data loss did not reset. 5-month-old infants seem to outperform 10-month-old infants, but data loss increased more sharply over the course of the experiment in the 5-month-old infants.

Fig. 5 shows the effect of the length of the task on data loss. The top panel shows data loss of trials throughout the entire continuous EEG experiment while the lower panel repeats this for the ERP experiment. Recall that during the continuous EEG experiment, six one-minute-long videos are presented. In between videos, each infant can take a short break as the new video is started. Fig. 5A reveals a clear effect of the breaks: they coincide with a decrease in data loss. A second finding is that there is only a minor upward trend indicating a higher likelihood of data loss over the course of the entire experiment. Comparison between the five- and ten-month-old infants further shows that both age groups perform rather similar over the course of the entire experiment.

Fig. 5B shows data loss as a function of the course of the task, for the ERP experiment. Every 24 trials mandatory breaks are taken by showing a short video clip (‘attention getter’). We can see the effect of these breaks, as a decrease in data loss right after the break. However, this effect appears more short-lived. Thus, the ERP experiment differs from the continuous experiment in that the upward trend in data loss proceeds shortly (almost immediately) after the break. Moreover, compared to the EEG experiment, the ERP experiment witnesses a more pronounced increase in data loss over the course of the experiment. It appears that both groups of infants react differently to the stimuli they are viewing. Differences between 5 and 10-month-old infants become smaller over the course of the ERP-experiment, but 5-month-old infants consistently show lower amounts of data loss than the 10-month-old infants do. Note that for both paradigms the first and last trials show a larger proportion of data loss, which possibly indicates an effect of starting and quitting the experiment.

### 3.3 Longitudinal effects and effects of entire study on data loss

We determined stability between 5 and 10-month-waves of several data loss characteristics by creating cross tables of these characteristics and performing a chi-square test for independence to check whether there is a relationship between the categorical variables. Table 3 is a cross table comparing the stability of infants who were either included or excluded due to fussiness over sessions. No significant relationship was found (*X*^2^(1, *N* = 757) = 1.28, *p* = n.s.).

**Table 3:** Cross table of included vs excluded based on fussiness.

		Session 2			
		Included		Excluded	
		N	%	N	%
Session 1	Included	515	86.7	128	82.1
	Excluded	86	14.3	28	17.9

Table 4 is a cross table of cap refusal of the 5-month-olds’ and 10-month-olds’ sessions. A significant relationship was found (*X*^2^(1, *N* = 980) = 4.4, *p* < .05), indicating that cap refusal in the first session influences the likelihood of cap refusal in session two and vice versa. Please note, however, that cap refusal is rare, and that only 3 / 980 infants refused the EEG cap in both sessions.

**Table 4:** Cross table of cap refusal.

		Session 2			
		Yes		No	
		N	%	N	%
Session 1	Yes	3	6.8	19	2.0
	No	41	93.2	917	98.0

Table 5 is a cross table for the amount of data loss. Data loss is categorized into high data loss (being in the group with the 50 % highest data loss) and low data loss (being in the group with the 50 % lowest data loss). A chi-square test for independence yielded a significant result (*X*^2^(1, *N* = 672) = 6.3, *p* < .05), indicating some stability over sessions for these categories. However, a correlation showed no relationship between individual values of data loss over both sessions (*r* = 0.070).

**Table 5:** Cross table of amount data loss.

		Session 2			
		Low		High	
		N	%	N	%
Session 1	Low	197	56.8	153	47.1
	High	150	43.2	172	52.9

## 4 Discussion

Attrition rates in infant EEG are usually high. While previous research has given a clear overview of which factors affect attrition rates similarly across studies, little is known about the factors contributing to data attrition within a study. In this paper, we showed that there is a wide array of factors influencing data attrition in one large-scale study. These factors can vary between subjects, possibly changing outcome measures and results, which may lead to biased conclusions.

The factors influencing data attrition described in this study can be broadly divided into three groups: child-related factors, testing-related factors, and longitudinal (study-specific) factors. Three child-related factors were found to influence data loss: gender, age, and head shape. Four testing-related factors were found to influence data loss: time of testing, the season of testing, research assistant present during the experiment, and task length all had considerable influence on data. Lastly, data attrition rates of the first session of testing were found to be related to the second session of testing, underlining possible longitudinal biases in terms of data loss. The influence of all these factors was found irrespective of which EEG task analyzed, even though data loss was found to be lower in the continuous EEG experiment presenting audio-visual video clips compared to the ERP paradigm presenting still images. Below we first discuss the main findings concerning our three factors of interest, then we will discuss the limitations and future directions of this study and lastly, we will formulate recommendations based on the results described here to minimize data loss in future infant EEG studies.

### 4.1 The impact of child-related factors on EEG data loss

By comparing data loss between the age groups in our study, we found a slight influence of age on data loss. The oldest group (three-year-old children) showed markedly lower data loss, but between 5- and 10-month-old infants, the difference in data loss was negligible. This is in contradiction with the finding of Slaughter and Suddendorf (2007), who described younger infants having higher slightly attrition rates compared to older infants, which they explained by a higher rate of sleepiness of younger infants during testing. What is likely is that in our study the higher likelihood of tiredness for younger infants during testing is offset by the higher likelihood of resistance to preparation procedures or restlessness in older infants (Hoehl and Wahl, 2012), resulting in similar data loss across waves. Support for this reasoning comes when we compare our results to data quality for the same set of children in a different session: eye tracking. Hessels and Hooge showed that for the eye-tracking sessions in the YOUth Infant & Child cohort there was a limited difference in data loss between 5 and 10-month-old infants, but that quality significantly improved for the three-year-olds (Hessels and Hooge, 2019).

Surprisingly, data loss was lower for boys than for girls. We did not anticipate this finding. We tentatively speculate post-hoc here that research assistants might find it easier to approach and handle boys, which speeds up capping, whereas they are more careful and considerate in capping girls. This fits with findings that mothers, too, behave differently with their daughters than with their sons (Clearfield and Nelson, 2006). A different possibility might have to do with differences in head circumference. Boys have generally larger heads during infancy (Niklasson and Albertsson-Wikland, 2008), which we also, on average, found in our study (boys: 44.8 cm +/-1.9, girls: 43.5 +/- 2.0). Larger heads could increase the fit of the cap and therefore decrease data loss.

Another factor we examined was the impact of infant head-shape. Irregularity of head shape causes a higher possibility of data loss in infants, which is probably caused by poorer cap fit. It is important to note that it is currently unknown how well this result generalizes towards other EEG equipment types. In our experience, irregular head shape of an infant affects data loss in electrodes towards the edges of the cap most negatively. This is likely caused by an increase or decrease in pressure on the electrode sites. Net-saline equipment could also be affected by this, but further research is necessary. For cap-gel equipment, this is evidence that the development of infant specialized EEG-equipment is warranted. One possible solution could be the creation of caps specifically designed for the most common head shape irregularities (flat-spotted back head, elongated head shape). While this increases costs in terms of material needed, this could be counterbalanced by a lower probability of data loss. Future testing is needed to understand whether creating caps for a wider range of head shapes increases data loss.

Finally, we asked whether parents’ judgments on the suitability of the child for this particular testing day affected data loss. We did not observe noticeable effects here: ‘regular days’ showed similar patterns as ‘days when children were judged to be tired’; or when parents reported that their child had just recovered from illness, like a cold. The most likely explanation for this effect is that infants who are too ill or too tired will not come in for testing and, as such, only slightly ill or tired infants participate. It is therefore essential that the lab is flexible and allows for rescheduling of appointments whenever the parents feel it is necessary. Small changes in the well-being of an infant will apparently not influence the likelihood of data loss.

### 4.2 The impact of measurement-related factors on EEG data loss

We also examined a wide range of factors regarding the testing arrangements. Above all findings, the choice of research assistant (RA) influenced data loss in our infant sample. The RA-dependency of infant EEG data underlines the importance of close monitoring of data loss. Even in the YOUth project, which has a rigorous training program for new RAs, with live-monitoring sessions, it is impossible to level the playing field between RAs in terms of data loss. In addition to the differences between RAs in data loss, a positive effect of experience was also found. Experience (as in the number of infants tested) was negatively related to data loss, both for the four RAs (Figure 6B) and for all RAs together.

A possible explanation for this effect of RA on data loss is that some RAs are more proficient in capping. Shortening capping times presumably enables children to have more energy and attention for what is coming next (i.e., the experiment), which enhances the likelihood of infants successfully completing the experiment. Note, however, there is no research yet examining the factor of capping time on data loss. Secondly, some RAs might prove better at calming both infants and parents in potentially stressful situations, resulting in less data loss. Also, some assistants might be more likely to intervene in the experiment when the EEG signal is deteriorating or when the child signals a need for a break (to e.g. eat a breadstick). Lastly, it is also possible that some RAs simply need more time to become proficient enough to limit data loss. Figure 6B hints at this with start data loss wildly differing between RAs, but all seemingly trending towards the same data loss limit. It is important to mention that none of the RAs tested here underperformed based on our expectations. We expected a dropout rate of 20–30 %, which all these RAs complied with. The main difference was caused by two overperforming RAs (data loss dropout rate of ≈ 10 %). Specifically studying the outperforming RAs could provide us with valuable information to limit future data loss.

What should be seen as a limiting factor is that studying the effect of RA on data loss is difficult as many other hidden factors could be driving differences in data loss. For example, some RAs could only have tested in the summer months, only during the early hours of the day, or only tested on certain weekdays. In our case, we only picked RAs who have tested for an extended period of time during the entirety of the study and are available throughout the day. So, the mentioned factors would likely not affect the outcome here, but there might be currently unknown factors that drive these differences. Therefore, it will be valuable to know how well this result translates to other studies.

A second factor that impacted data loss was the time of onset of the EEG task. Earlier hunches were confirmed that early morning testing lowered data loss. This is likely to be caused by early morning testing fitting better in most infants’ sleep/eat-schedules, with most young infants waking and eating early. An early morning participant will, therefore, more likely to be well-rested and well-fed. Also, an early morning participant will have had fewer chances to be overwhelmed by experiences that are out of the ordinary.

Besides the time of day, we also found an influence of the season in which testing took place. Infants generally performing better during the warmer months in The Netherlands (spring and summer). One possible explanation for this is that during the warmer months in The Netherlands, infants have a possible lower likelihood of colds or flu. Even when parents indicate that their child can participate even though he or she just recovered from an illness such as the flu, it is our experience that these children can be irritable and do not tolerate capping. A different explanation of why data loss is lower in spring and summer could be related to the higher humidity during the summer months. Humidity affects skin impedance during a measurement (Clar et al., 1975). Therefore, higher humidity can increase the ease of signal transmission between the electrode and the scalp, lowering the possibility of data loss. Opposingly, an increase in high humidity can also increase sweating, which in turn can cause sweating artifacts (White and Van Cott, 2015).

Lastly, the factor of time elapsed during a task impacted data loss. The more the task progressed, the higher the likelihood there was data loss. This effect was heightened in the ERP design, which showed a clear increase in data loss over the course of the experiment. These results are in line with earlier studies, which found results from ERP studies to change over the course of an experiment (Nikkel and Karrer, 2009; Stets et al., 2012). Both of our experiments revealed a positive effect of breaks: with the continuous experiment resetting in data loss likelihood after every break while the ERP experiment only witnessed a short improvement in chance on data loss, after which it reverted to the original data loss progression. This could be caused by infants getting habituated to the ERP paradigm. The ERP paradigm has little changes over the course of the experiment, while the continuous EEG paradigm changes constantly over the course of every video. This underlines the importance of the development of new EEG paradigms specifically designed for infants. It is vital to not only consider the choice of stimuli but also how to implement new ways to take breaks. In the continuous experiment, the restarting of a new video seemed to have a desirable effect, while the showing of a short movie clip did not cause a similar long-lived effect in the ERP experiment.

### 4.3 The impact of longitudinal factors on EEG data loss

Some evidence was found for stability of data loss over waves. Data attrition due to fussiness was not found to be stable over sessions. However, infants who were categorized as high data loss in one session were more likely to be categorized similarly in the other session and vice versa. This ties in with the earlier found relationship between infant temperament and data attrition (Marshall et al., 2009), where infants with a negative temperament showed higher attrition rates than infants with a positive temperament. This could prove problematic in longitudinally designed studies, as infants with certain character traits would have biases in data availability. This might influence outcomes and in turn, can lead us to draw wrong conclusions when comparing infants. Moreover, it questions whether all results can be generalizable to the whole population.

Similar results are found in cap refusal: when an infant refused to participate in the experiment in one session, he or she was more likely to refuse to participate in the other session as well. This result might be used for future researchers to decide to call on an infant who has refused to participate in a study before since there is a higher likelihood of cap refusal. What is important to note, however, is that cap refusal in our study was exceedingly rare. So rare that only 3 infants (of the in total 980 tested) refused the cap twice.

### 4.4 Limitations of the current study

Some caveats have to be taken into account when judging the abovementioned conclusions. First and foremost, we studied each factor in isolation. As a result, the exact relationship between the interplay of factors described here and data loss remains difficult to characterize. Moreover, there may be other, more fundamental, factors affecting the factors described here. Perhaps the differences between RAs are all to be explained if we realize that some RAs frequently start at 10 a.m. or whether some RAs only work on some workdays. We have checked these specifically and found them to be untrue, but there could be countless other factors influencing data loss, stretching beyond our current imagination. Therefore, while our results suggest that some factors can explain data loss, these factors may share a common origin currently unknown.

Secondly, this study focuses only on the external factors related to infant and setting but ignores any factors related to technical issues arising from using EEG in infants. For instance, the two most popular techniques currently used are cap-gel and net-saline systems. Since our study only uses cap-gel equipment, we cannot systematically compare these techniques on data loss (DeBoer et al., 2007; Johnson et al., 2001). Readers, therefore, have to be careful generalizing these results to other EEG techniques. This is especially true for head shape, which could have different effects on net-saline equipment. Also, researchers vary widely in how they pre-process the data (e.g., in choice of reference electrode; filtering, and trial rejection criteria). We did not manipulate the choice of analyses here since we felt this would go beyond the scope of the paper. There are other papers available that examine the consequences of different pre-processing steps or analyses (Luck, 2010). Future research is warranted to examine whether these technical issues influence infant EEG data loss differently.

Lastly, it might be tempting to equate data loss to data quality, but it is not. Future research is necessary to better understand what constitutes acceptable data for different tasks. On the one hand, previous studies have found that studying more infants with fewer trials can sometimes yield better and more reliable results in infant ERP studies (Stahl et al., 2010; Stets and Reid, 2011). On the other hand, in face-processing ERP tasks, ERPs appear more reliable when more trials are included (Munsters et al., 2019). For EEG connectivity, current evidence seems to point to more data equates to more reliable results (Fraschini et al., 2016; van der Velde et al., 2019), but it is unknown whether this always holds true. Recall that there is no such thing as a golden standard for minimum amount of required data in an EEG study. Therefore, future studies are warranted to increase our understanding in which cases, which factors have to be taken into account to increase result replicability and quality.

### 4.5 General recommendations

Our results presented here, in addition with the earlier studies on data loss and attrition in infant EEG (Hoehl and Wahl, 2012; Slaughter and Suddendorf, 2007; Stets et al., 2012), lead us to provide the following recommendations for developmental EEG researchers. These recommendations can hopefully be used in future studies to ease the gathering and analysis of infant EEG data.

Firstly, it is important to understand that there is a wide range of factors that potentially influence data loss in infant EEG studies. Some of these factors are well-established factors, like the length of the experiment, age, or child temperament, but there are also external influences that researchers are less likely able to control, like the season of testing or infant gender. It is especially important to understand that these uncontrollable external factors could be represented in your different groups in a biased way. For example, in a longitudinal study, one could study a group of young infants in the summer and then re-test them 6 months later in the winter. This could increase data loss specifically in the re-test group, possibly biasing outcomes in comparing the two groups. Also, this could lead to increased data attrition, limiting the power of your longitudinal analysis.

It is likely that a far wider range of factors than described in this study influence data loss and data attrition. Keeping this in mind when analyzing studies is vital, especially with data-driven approaches, like Hidden Markov Models and machine learning. Such factors potentially could mask our understanding of why differences between groups exist. Differences can likely arise from differences in data loss due to these external factors. Either from biases in the representation of certain infant characteristics or through lower reliability of measures gathered with increased data loss. Keeping a running tally of data loss and attrition can aid one’s understanding of when and why certain important events happen.

Secondly, when studying the infant brain through EEG, we advise limiting the use of different research assistants. Not only did we observe influences from the research assistant present during testing on the amount of data loss, but we also saw that experience greatly reduced variation across RAs coupled with an overall decrease in data loss. In large-scale studies (like the YOUth project) it is not always possible to rely on a small set of RAs. In these cases, closely monitor your assistants. In the YOUth project, we have set up a running tally of data loss for each assistant. This allows a better understanding of which conditions data loss happens and also to intervene if necessary. More importantly, it allows us to learn from those assistants who are exceeding our expectations.

Thirdly, it is important to understand that controlling for data loss is not always feasible. If data loss is biased in the amount between your groups, controlling for it could hide the true outcome. Moreover, increased data loss might lead to increased attrition. Having to test extra infants in certain groups to ensure equal population sizes could bias certain groups by increasing the likelihood of infants with better temperament being in certain groups. Therefore, ensuring environmental factors are least likely to cause data loss should be one of the most important points in designing infant EEG studies. We summarize these points as follows:

1 Focus on testing early in the day, paying heed to infant eat and sleep schedules.
2 Preferably test in the summer or spring months.
3 Keep the experiment short and interesting. Create infant-specific stimuli, with breaks. Use auditory (or audio-visual) stimuli if possible.
4 Limit the number of research assistants used and designate them to EEG recordings only if possible

Finally, it is unknown how well the results described here generalize to other infant EEG studies. We believe we can only advance our understanding of what factors contribute to data loss when more studies are more explicit in reporting their numbers of attrition and data loss. This echoes the recommendations put forward in the meta-analysis on data attrition by Stets and colleagues (2012), who reported that many studies prior to 2012 did not include such information. Failure to include these types of information limits our understanding of the origins and reasons for data loss. Moreover, it could mask biases within data sets. We, therefore, recommend that studies should report attrition rates split, at least, by age groups and gender. Ideally, to further improve general insights into the data loss of infant EEG studies, studies should include data loss distributions to visualize differences or highlight potential biases between groups.

These recommendations are not only meant for researchers, but also for editors and reviewers of developmental journals. The field requires new guidelines to which infant EEG researchers need to adhere to when publishing their data. Especially, as data sets become larger and cross-lab collaborations increasingly common, we need better insights into the quality and biases in which individual data sets are collected.

## 5 Conclusions

Data loss in infant EEG is costly, as it leads to underpowered infant EEG studies. One (undesirable) solution to add power would be to test more infants, but this requires time, money, and easy access to infants. Another solution would be to create a testing environment that limits data loss. It is therefore of the utmost importance to design infant EEG studies specifically with limiting data loss in mind. Many decisions that researchers make to limit data loss are based on their hunches. This study put these hunches and other factors to the test, by comparing data loss distribution across several factors related to the setting or the infant itself. These factors have to be kept in mind when designing and analyzing new infant EEG studies. We hope that this article not only informs the reader but also progresses the debate on the topic of EEG data loss in infants.

## Declaration of Competing Interest

The authors declare that they have no known competing financial interests or personal relationships that could have appeared to influence the work reported in this paper.

## Footnotes

1 Using *ft_artifact_jump* with standard options
