# Supplementary material for: Limiting data loss in infant EEG: putting hunches to the test
Source: Dev Cogn Neurosci. 2020 Jun 26;45:100809. doi: 10.1016/j.dcn.2020.100809 (PMC7358181; doi:10.1016/j.dcn.2020.100809)
Supplement: Supplementary file 1 [file mmc1.docx]

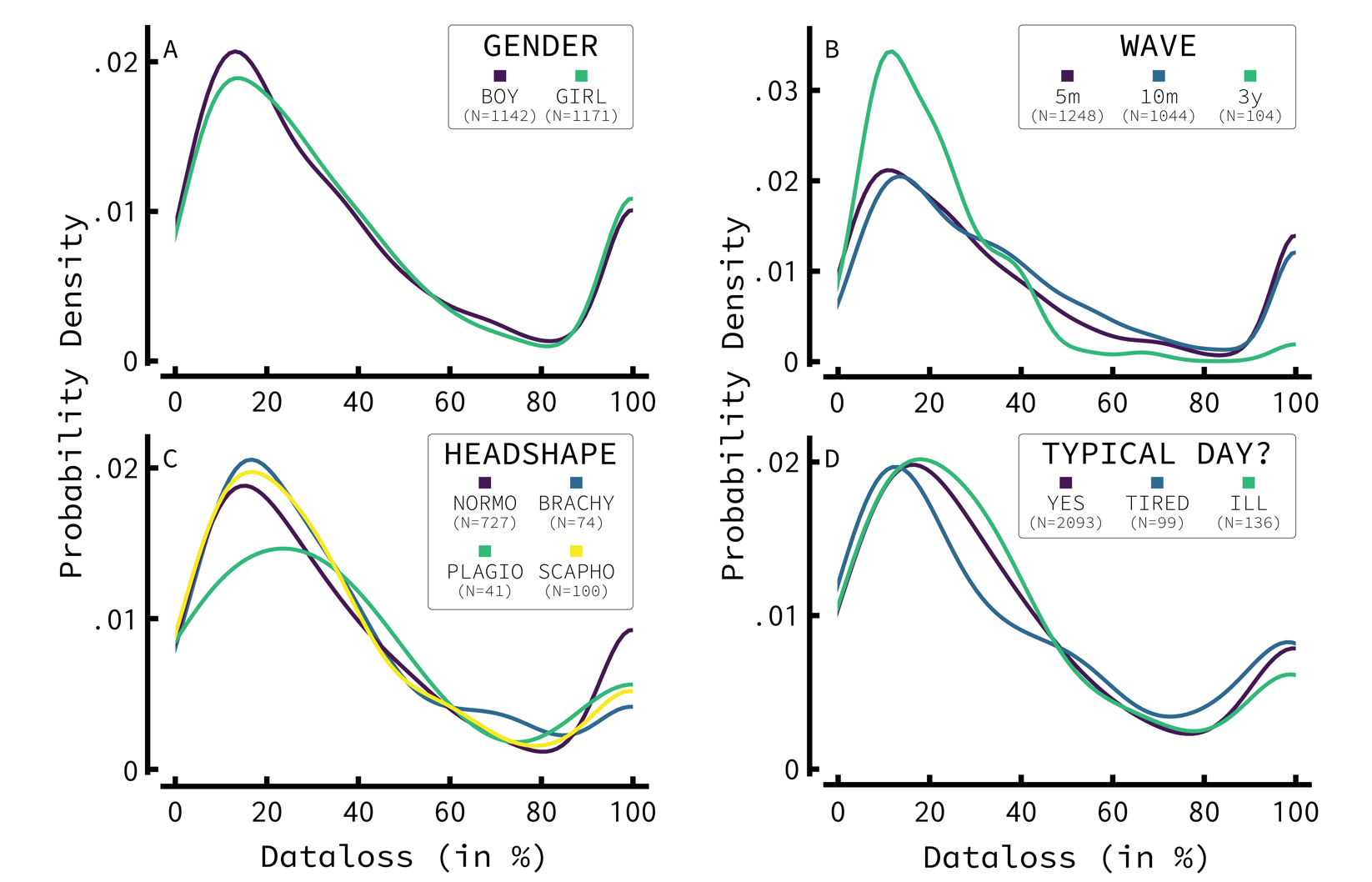


**Supplementary figure 1. Effects of infant-related external factors on ERP data quality.** Plots with data loss distributions of all external factors also tested with continuous EEG data. Gender, wave, head shape, and typical day all show similar plots as with continuous EEG data, underlining the influence of these factors on data quality.


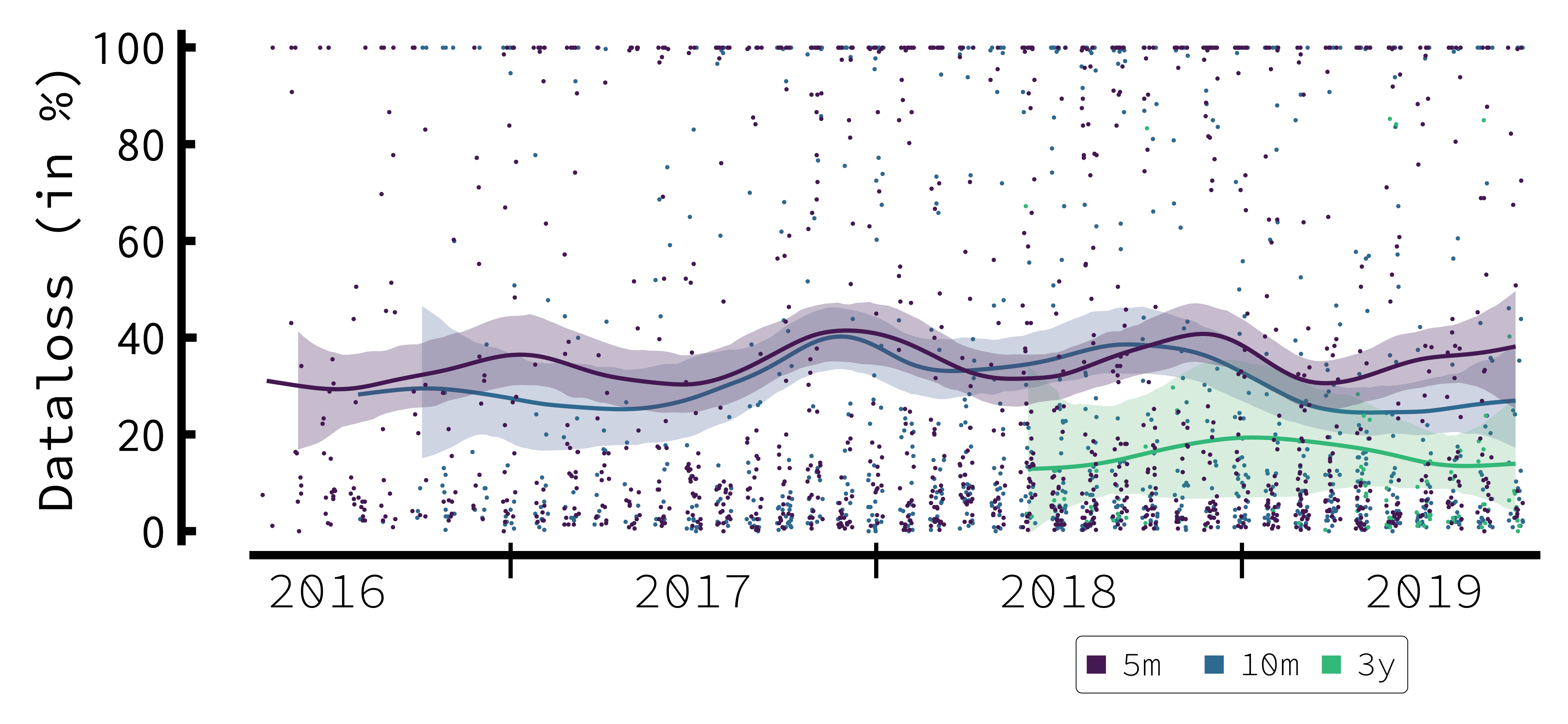


**Supplementary figure 2. Data loss over the course of the entire study.** Data loss over the course of the entire study is shown here. Every dot is a measurement of a single subject in a particular month. Data is slightly jittered to increase visibility of most dots. A smoothed line of the averages per month is shown here. A cyclic rhythm can be seen with lower data loss in the spring and summer months (middle of each year) and higher data loss at the start and towards the end of each year (winter and autumn). This rhythm can be found across age groups.


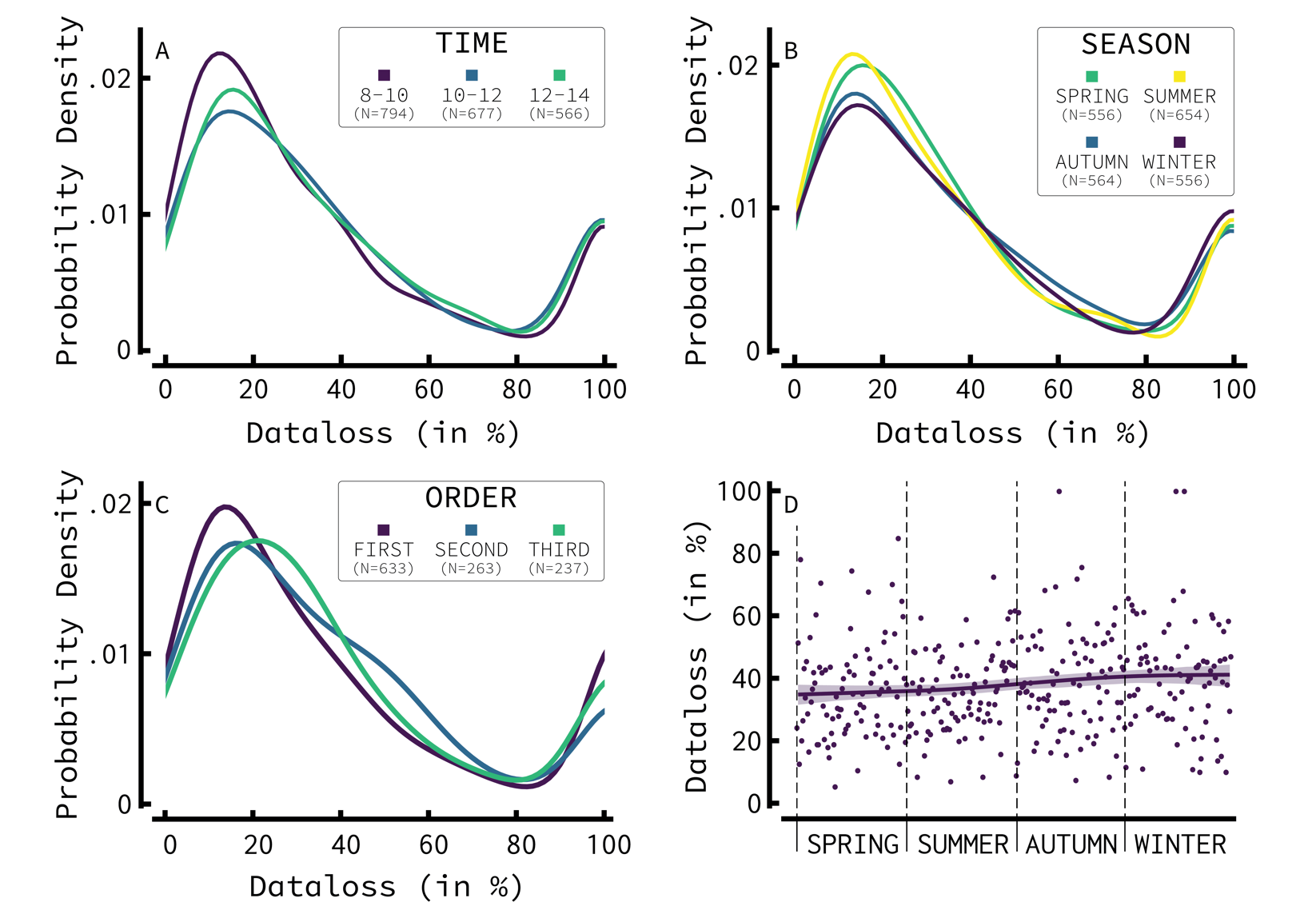


**Supplementary figure 3. Effects of timing-related external factors on ERP data quality.** Plots with data loss distributions of all external factors also tested with continuous EEG data. Time of onset of testing, order, and season all show great overlap with the continuous EEG plots. Once again, a distinct bump in data loss can be seen in figure 2D) around autumn and winter.
